# Supplementary material for: Deletion of wfs1 Impairs Oligodendrocyte Precursor Cells Dorsal Distribution and Myelination Through the wfs1-hmgcs1 Axis in Zebrafish
Source: Biology (Basel). 2026 Aug 16;15(16):1402. doi: 10.3390/biology15161402 (PMC13510151; doi:10.3390/biology15161402)
Supplement: Supplementary file 1 [file biology-15-01402-s001.zip › File S1-Transcriptome sequencing data and differentially expressed genes (DEGs) in wfs1-- zebrafish at 5 dpf/2.KEGG/wfs1KOvsWT/GSEA/WT/APOPTOSIS/APOPTOSIS(DRE04210).html]

Details for gene set APOPTOSIS(DRE04210)[GSEA]

|  || Dataset | deg.sample |
| Phenotype | sample.cls |
| Upregulated in class | WT |
| GeneSet | APOPTOSIS(DRE04210) |
| Enrichment Score (ES) | -0.23934862 |
| Normalized Enrichment Score (NES) | -1.3198406 |
| Nominal p-value | 0.026209677 |
| FDR q-value | 0.5863229 |
| FWER p-Value | 0.956 |
Table: GSEA Results Summary

  

Fig 1: Enrichment plot: APOPTOSIS(DRE04210)      
 Profile of the Running ES Score & Positions of GeneSet Members on the Rank Ordered List

  

| PROBE | DESCRIPTION (from dataset) | GENE SYMBOL | GENE\_TITLE | RANK IN GENE LIST | RANK METRIC SCORE | RUNNING ES | CORE ENRICHMENT || 1 | 57934\_1 | actb1 |  |  | 1 | 4.982 | 0.0299 | No |
| 2 | 567021 | ern2 |  |  | 181 | 4.340 | 0.0503 | No |
| 3 | 570238 | fosaa |  |  | 589 | 2.782 | 0.0542 | No |
| 4 | 561021 | si:dkey-103e21.5 |  |  | 599 | 2.768 | 0.0706 | No |
| 5 | 431763 | gadd45ab |  |  | 713 | 2.560 | 0.0824 | No |
| 6 | 100000252\_3 | pik3r3b |  |  | 716 | 2.550 | 0.0977 | No |
| 7 | 556410\_1 | atf4b |  |  | 780 | 2.425 | 0.1102 | No |
| 8 | 58133 | ngfb |  |  | 1219 | 1.881 | 0.1077 | No |
| 9 | 569298 | ctsbb |  |  | 1451 | 1.690 | 0.1106 | No |
| 10 | 768248 | fas |  |  | 1486 | 1.661 | 0.1195 | No |
| 11 | 101886621\_1 | LOC101886621 |  |  | 1602 | 1.594 | 0.1254 | No |
| 12 | 394198 | fosab |  |  | 1745 | 1.508 | 0.1300 | No |
| 13 | 100135062\_2 | csf2rb |  |  | 1809 | 1.460 | 0.1368 | No |
| 14 | 65225 | ctsd |  |  | 1835 | 1.441 | 0.1447 | No |
| 15 | 552927 | casp6 |  |  | 1941 | 1.394 | 0.1497 | No |
| 16 | 100000252 | pik3r3b |  |  | 1965 | 1.382 | 0.1573 | No |
| 17 | 791531 | casp6l1 |  |  | 2037 | 1.353 | 0.1632 | No |
| 18 | 431777 | tuba7l |  |  | 2071 | 1.338 | 0.1702 | No |
| 19 | 373080 | tuba1b |  |  | 2072 | 1.337 | 0.1782 | No |
| 20 | 559564\_1 | eif2ak3 |  |  | 2076 | 1.336 | 0.1862 | No |
| 21 | 445091\_1 | zgc:92480 |  |  | 2191 | 1.289 | 0.1903 | No |
| 22 | 566604 | casp3b |  |  | 2287 | 1.253 | 0.1949 | No |
| 23 | 373107 | birc2 |  |  | 2293 | 1.250 | 0.2022 | No |
| 24 | 563560 | ikbkb |  |  | 2417 | 1.202 | 0.2056 | No |
| 25 | 393548 | gadd45aa |  |  | 2430 | 1.197 | 0.2124 | No |
| 26 | 30590\_1 | tp53 |  |  | 2944 | 1.051 | 0.2025 | No |
| 27 | 497646 | gadd45bb |  |  | 3089 | 1.017 | 0.2041 | No |
| 28 | 573216 | tuba1a |  |  | 3240 | 0.984 | 0.2052 | No |
| 29 | 393317 | diabloa |  |  | 3306 | 0.971 | 0.2090 | No |
| 30 | 567611 | itpr1a |  |  | 3360 | 0.962 | 0.2131 | No |
| 31 | 564295\_1 | traf2a |  |  | 3833 | 0.862 | 0.2034 | No |
| 32 | 337223 | tuba8l |  |  | 3910 | 0.846 | 0.2061 | No |
| 33 | 404211 | pik3r2 |  |  | 4059 | 0.817 | 0.2063 | No |
| 34 | 565825 | si:dkeyp-50d11.2 |  |  | 4870 | 0.698 | 0.1849 | No |
| 35 | 569326 | zgc:174855 |  |  | 4934 | 0.689 | 0.1871 | No |
| 36 | 100004321 | LOC100004321 |  |  | 5124 | 0.665 | 0.1851 | No |
| 37 | 564295 | traf2a |  |  | 5504 | 0.634 | 0.1769 | No |
| 38 | 436641 | ctsl.1 |  |  | 5534 | 0.633 | 0.1798 | No |
| 39 | 795573 | prf1.6 |  |  | 5547 | 0.632 | 0.1833 | No |
| 40 | 323099 | nfkbiab |  |  | 5568 | 0.630 | 0.1864 | No |
| 41 | 564921 | bada |  |  | 5627 | 0.623 | 0.1883 | No |
| 42 | 559384 | prf1.5 |  |  | 5725 | 0.614 | 0.1890 | No |
| 43 | 751765 | pmaip1 |  |  | 5838 | 0.604 | 0.1891 | No |
| 44 | 58100 | badb |  |  | 5892 | 0.598 | 0.1910 | No |
| 45 | 558237 | dab2ipb |  |  | 5984 | 0.589 | 0.1916 | No |
| 46 | 735295 | faslg |  |  | 6149 | 0.572 | 0.1899 | No |
| 47 | 556486 | sept4a |  |  | 6511 | 0.536 | 0.1817 | No |
| 48 | 406304 | gadd45ba |  |  | 6674 | 0.522 | 0.1797 | No |
| 49 | 567046 | cts12 |  |  | 6895 | 0.500 | 0.1758 | No |
| 50 | 402991 | gadd45ga |  |  | 6915 | 0.499 | 0.1782 | No |
| 51 | 65236 | mapk8b |  |  | 6998 | 0.492 | 0.1786 | No |
| 52 | 58130 | tradd |  |  | 7033 | 0.489 | 0.1804 | No |
| 53 | 792024 | si:ch73-199e17.1 |  |  | 7117 | 0.481 | 0.1807 | No |
| 54 | 561737 | pik3ca |  |  | 7195 | 0.474 | 0.1811 | No |
| 55 | 100005496 | si:ch211-1i11.3 |  |  | 7276 | 0.468 | 0.1814 | No |
| 56 | 393154 | tuba8l4 |  |  | 7289 | 0.467 | 0.1838 | No |
| 57 | 335916 | jun |  |  | 7293 | 0.467 | 0.1865 | No |
| 58 | 373110 | birc5a |  |  | 7442 | 0.457 | 0.1846 | No |
| 59 | 554157 | ctss2.1 |  |  | 7792 | 0.438 | 0.1762 | No |
| 60 | 100333521 | LOC100333521 |  |  | 7991 | 0.424 | 0.1725 | No |
| 61 | 558045 | parp4 |  |  | 8200 | 0.415 | 0.1684 | No |
| 62 | 562585 | itpr2 |  |  | 8204 | 0.415 | 0.1708 | No |
| 63 | 560883 | ptpn13 |  |  | 8737 | 0.385 | 0.1564 | No |
| 64 | 100005496\_1 | si:ch211-1i11.3 |  |  | 8818 | 0.380 | 0.1561 | No |
| 65 | 373118 | casp2 |  |  | 8924 | 0.373 | 0.1550 | No |
| 66 | 58122 | mcl1a |  |  | 8998 | 0.368 | 0.1549 | No |
| 67 | 406463 | nfkbiaa |  |  | 9066 | 0.363 | 0.1550 | No |
| 68 | 768202 | pdpk1a |  |  | 9086 | 0.361 | 0.1566 | No |
| 69 | 378972 | akt2 |  |  | 9293 | 0.347 | 0.1522 | No |
| 70 | 373108 | xiap |  |  | 9360 | 0.343 | 0.1521 | No |
| 71 | 100002454 | dab2ipa |  |  | 9672 | 0.323 | 0.1443 | No |
| 72 | 406507 | gadd45gb.1 |  |  | 9742 | 0.319 | 0.1440 | No |
| 73 | 393664 | tuba8l2 |  |  | 9767 | 0.318 | 0.1451 | No |
| 74 | 100331324 | LOC100331324 |  |  | 9791 | 0.316 | 0.1463 | No |
| 75 | 562720 | mapk8a |  |  | 9888 | 0.310 | 0.1451 | No |
| 76 | 327377 | tuba4l |  |  | 9990 | 0.304 | 0.1438 | No |
| 77 | 557526 | traf2b |  |  | 10340 | 0.282 | 0.1345 | No |
| 78 | 321564 | eif2s1b |  |  | 10470 | 0.275 | 0.1320 | No |
| 79 | 558045\_1 | parp4 |  |  | 10679 | 0.262 | 0.1270 | No |
| 80 | 393228\_1 | zgc:55558 |  |  | 10754 | 0.257 | 0.1262 | No |
| 81 | 563181 | map2k2a |  |  | 10792 | 0.256 | 0.1266 | No |
| 82 | 795766 | LOC795766 |  |  | 11495 | 0.217 | 0.1057 | No |
| 83 | 405785 | tnfa |  |  | 11720 | 0.205 | 0.0999 | No |
| 84 | 565588 | ctsf |  |  | 11927 | 0.194 | 0.0946 | No |
| 85 | 559425 | bida |  |  | 12011 | 0.190 | 0.0931 | No |
| 86 | 556410\_2 | atf4b |  |  | 12071 | 0.187 | 0.0923 | No |
| 87 | 406645 | ctsba |  |  | 12298 | 0.174 | 0.0863 | No |
| 88 | 324818 | ctsh |  |  | 12480 | 0.165 | 0.0815 | No |
| 89 | 571033 | pidd1 |  |  | 12588 | 0.160 | 0.0791 | No |
| 90 | 557302 | casp8l2 |  |  | 12635 | 0.157 | 0.0786 | No |
| 91 | 195815 | lmna |  |  | 12787 | 0.150 | 0.0747 | No |
| 92 | 559849 | prf1.3 |  |  | 12976 | 0.142 | 0.0697 | No |
| 93 | 560788 | parp1 |  |  | 13023 | 0.139 | 0.0690 | No |
| 94 | 100049706 | casp8l1 |  |  | 13088 | 0.136 | 0.0678 | No |
| 95 | 570425 | diablob |  |  | 13472 | 0.117 | 0.0564 | No |
| 96 | 30196 | lmnb2 |  |  | 13734 | 0.105 | 0.0488 | No |
| 97 | 195816 | lmnb1 |  |  | 13738 | 0.105 | 0.0494 | No |
| 98 | 114401 | bcl2l1 |  |  | 13894 | 0.097 | 0.0450 | No |
| 99 | 569698 | mapk10 |  |  | 13907 | 0.096 | 0.0452 | No |
| 100 | 100149794 | akt3b |  |  | 14249 | 0.080 | 0.0350 | No |
| 101 | 100149388 | itpr1b |  |  | 14261 | 0.080 | 0.0351 | No |
| 102 | 565756 | nfkb1 |  |  | 14558 | 0.067 | 0.0261 | No |
| 103 | 30443 | ctslb |  |  | 14633 | 0.064 | 0.0242 | No |
| 104 | 58081 | baxa |  |  | 14773 | 0.057 | 0.0201 | No |
| 105 | 58022 | casp8 |  |  | 15145 | 0.039 | 0.0087 | No |
| 106 | 100007906\_1 | parp2 |  |  | 15576 | 0.018 | -0.0048 | No |
| 107 | 30380 | nras |  |  | 15635 | 0.015 | -0.0066 | No |
| 108 | 445164 | tuba8l3 |  |  | 15740 | 0.009 | -0.0098 | No |
| 109 | 563053 | capn2b |  |  | 15938 | 0.001 | -0.0160 | No |
| 110 | 564835 | si:dkey-269i1.4 |  |  | 16122 | -0.006 | -0.0218 | No |
| 111 | 791141 | zgc:158659 |  |  | 16140 | -0.006 | -0.0223 | No |
| 112 | 565825\_1 | si:dkeyp-50d11.2 |  |  | 16172 | -0.008 | -0.0232 | No |
| 113 | 100005446 | LOC100005446 |  |  | 16477 | -0.017 | -0.0327 | No |
| 114 | 336746 | zgc:63831 |  |  | 16592 | -0.020 | -0.0362 | No |
| 115 | 373125\_1 | aifm1 |  |  | 16604 | -0.020 | -0.0364 | No |
| 116 | 570772 | bcl2a |  |  | 16638 | -0.021 | -0.0373 | No |
| 117 | 373130 | dffb |  |  | 16787 | -0.026 | -0.0418 | No |
| 118 | 100148503 | traf1 |  |  | 16862 | -0.029 | -0.0440 | No |
| 119 | 100001210 | si:ch211-165b19.9 |  |  | 17040 | -0.034 | -0.0494 | No |
| 120 | 564403 | si:ch211-153j24.3 |  |  | 17386 | -0.049 | -0.0600 | No |
| 121 | 335495 | parp3 |  |  | 17558 | -0.056 | -0.0650 | No |
| 122 | 100149273 | mapk9 |  |  | 17865 | -0.070 | -0.0743 | No |
| 123 | 394174 | pik3cd |  |  | 17974 | -0.074 | -0.0773 | No |
| 124 | 30270 | ngfa |  |  | 17981 | -0.075 | -0.0770 | No |
| 125 | 450022 | ctsz |  |  | 18182 | -0.084 | -0.0828 | No |
| 126 | 58131 | apaf1 |  |  | 18211 | -0.086 | -0.0832 | No |
| 127 | 767645 | zgc:153993 |  |  | 18516 | -0.099 | -0.0922 | No |
| 128 | 404211\_1 | pik3r2 |  |  | 18555 | -0.101 | -0.0928 | No |
| 129 | 415099 | rela |  |  | 18848 | -0.116 | -0.1013 | No |
| 130 | 553383 | spna2 |  |  | 19315 | -0.136 | -0.1152 | No |
| 131 | 560549 | akt3a |  |  | 19324 | -0.136 | -0.1146 | No |
| 132 | 399480 | mapk3 |  |  | 19514 | -0.144 | -0.1197 | No |
| 133 | 406728 | map2k1 |  |  | 19540 | -0.145 | -0.1197 | No |
| 134 | 337730\_1 | capn8 |  |  | 19702 | -0.153 | -0.1238 | No |
| 135 | 58130\_1 | tradd |  |  | 19801 | -0.157 | -0.1260 | No |
| 136 | 541374 | capn2a |  |  | 19921 | -0.163 | -0.1288 | No |
| 137 | 30546 | ntrk1 |  |  | 20060 | -0.169 | -0.1321 | No |
| 138 | 557109 | raf1b |  |  | 20203 | -0.175 | -0.1355 | No |
| 139 | 406471 | tnfrsf1a |  |  | 20265 | -0.178 | -0.1364 | No |
| 140 | 567046\_1 | cts12 |  |  | 20588 | -0.193 | -0.1454 | No |
| 141 | 373093 | hdr |  |  | 20727 | -0.200 | -0.1486 | No |
| 142 | 561924 | ddit3 |  |  | 20880 | -0.208 | -0.1521 | No |
| 143 | 100003446 | sept4b |  |  | 21118 | -0.222 | -0.1583 | No |
| 144 | 337572 | ctss2.2 |  |  | 21475 | -0.240 | -0.1681 | No |
| 145 | 436866 | tnfsf10 |  |  | 21725 | -0.253 | -0.1744 | No |
| 146 | 195817 | lmnl3 |  |  | 21861 | -0.260 | -0.1771 | No |
| 147 | 541530 | ikbkg |  |  | 21943 | -0.264 | -0.1781 | No |
| 148 | 368704 | ctsc |  |  | 22041 | -0.269 | -0.1795 | No |
| 149 | 373114 | cflara |  |  | 22061 | -0.270 | -0.1785 | No |
| 150 | 406303 | tuba2 |  |  | 22113 | -0.274 | -0.1785 | No |
| 151 | 403064 | atm |  |  | 22311 | -0.285 | -0.1830 | No |
| 152 | 559425\_1 | bida |  |  | 22391 | -0.289 | -0.1837 | No |
| 153 | 100190951 | bcl2l11 |  |  | 22410 | -0.290 | -0.1826 | No |
| 154 | 567460 | ripk1l |  |  | 23090 | -0.333 | -0.2020 | No |
| 155 | 360144\_1 | mapk1 |  |  | 23446 | -0.357 | -0.2111 | No |
| 156 | 100002263 | pik3cb |  |  | 23508 | -0.361 | -0.2108 | No |
| 157 | 795591 | si:ch211-114n24.6 |  |  | 23557 | -0.364 | -0.2102 | No |
| 158 | 445091 | zgc:92480 |  |  | 23577 | -0.365 | -0.2086 | No |
| 159 | 557176 | pik3r1 |  |  | 23610 | -0.367 | -0.2074 | No |
| 160 | 564906 | MGC174155 |  |  | 23623 | -0.368 | -0.2055 | No |
| 161 | 565106 | capn1b |  |  | 23645 | -0.370 | -0.2040 | No |
| 162 | 567333 | ctso |  |  | 23736 | -0.376 | -0.2045 | No |
| 163 | 100001785 | si:dkey-239j18.3 |  |  | 23826 | -0.382 | -0.2051 | No |
| 164 | 100001138 | si:ch211-165b19.8 |  |  | 23868 | -0.384 | -0.2041 | No |
| 165 | 65225\_2 | ctsd |  |  | 23976 | -0.390 | -0.2051 | No |
| 166 | 567611\_1 | itpr1a |  |  | 24145 | -0.403 | -0.2080 | No |
| 167 | 393417 | capn1a |  |  | 24498 | -0.419 | -0.2166 | No |
| 168 | 795573\_1 | prf1.6 |  |  | 24532 | -0.420 | -0.2151 | No |
| 169 | 567623 | zgc:174153 |  |  | 24679 | -0.428 | -0.2171 | No |
| 170 | 100002225 | map2k2b |  |  | 24736 | -0.433 | -0.2163 | No |
| 171 | novel.939 | - |  |  | 24769 | -0.436 | -0.2147 | No |
| 172 | 393287 | chuk |  |  | 25038 | -0.449 | -0.2204 | No |
| 173 | 569443\_1 | prf1.9 |  |  | 25116 | -0.454 | -0.2202 | No |
| 174 | 492763 | casp9 |  |  | 25122 | -0.455 | -0.2176 | No |
| 175 | 65225\_1 | ctsd |  |  | 25158 | -0.457 | -0.2159 | No |
| 176 | 415158 | cycsb |  |  | 25213 | -0.461 | -0.2149 | No |
| 177 | 553353 | si:ch211-202f3.3 |  |  | 25417 | -0.477 | -0.2184 | No |
| 178 | 57935 | actb2 |  |  | 25530 | -0.487 | -0.2190 | No |
| 179 | 449826\_1 | ctsll |  |  | 25788 | -0.510 | -0.2241 | No |
| 180 | 30716 | raf1a |  |  | 25882 | -0.518 | -0.2239 | No |
| 181 | 403000 | pdpk1b |  |  | 26060 | -0.534 | -0.2263 | No |
| 182 | 562317 | fadd |  |  | 26068 | -0.535 | -0.2233 | No |
| 183 | 768248\_1 | fas |  |  | 26075 | -0.536 | -0.2203 | No |
| 184 | 406768 | zgc:55262 |  |  | 26113 | -0.538 | -0.2182 | No |
| 185 | 321807 | eif2s1a |  |  | 26238 | -0.551 | -0.2188 | No |
| 186 | 550286 | hrasa |  |  | 26472 | -0.575 | -0.2227 | No |
| 187 | 794666\_1 | itpr3 |  |  | 26950 | -0.625 | -0.2340 | No |
| 188 | 564979 | wu:fa26c03 |  |  | 27120 | -0.641 | -0.2355 | Yes |
| 189 | 100000986 | gzm3 |  |  | 27226 | -0.645 | -0.2349 | Yes |
| 190 | 100002293 | endog |  |  | 27230 | -0.645 | -0.2311 | Yes |
| 191 | 555703\_1 | map3k5 |  |  | 27481 | -0.671 | -0.2350 | Yes |
| 192 | 103911769 | LOC103911769 |  |  | 27510 | -0.674 | -0.2318 | Yes |
| 193 | 553656 | hrasb |  |  | 27554 | -0.680 | -0.2291 | Yes |
| 194 | 321453 | ctsla |  |  | 27670 | -0.696 | -0.2286 | Yes |
| 195 | 100537591 | si:dkey-269i1.3 |  |  | 27698 | -0.701 | -0.2252 | Yes |
| 196 | 100135062 | csf2rb |  |  | 27817 | -0.719 | -0.2246 | Yes |
| 197 | 555703 | map3k5 |  |  | 27827 | -0.719 | -0.2206 | Yes |
| 198 | 100004261 | map3k14a |  |  | 28122 | -0.759 | -0.2253 | Yes |
| 199 | 393398 | ctss1 |  |  | 28315 | -0.789 | -0.2266 | Yes |
| 200 | 101910198 | akt1 |  |  | 28441 | -0.810 | -0.2257 | Yes |
| 201 | 558237\_1 | dab2ipb |  |  | 28671 | -0.851 | -0.2278 | Yes |
| 202 | 406514 | atf4a |  |  | 28789 | -0.876 | -0.2263 | Yes |
| 203 | 100536033 | LOC100536033 |  |  | 29056 | -0.934 | -0.2290 | Yes |
| 204 | 553634 | casp7 |  |  | 29151 | -0.959 | -0.2262 | Yes |
| 205 | 436904 | dffa |  |  | 29210 | -0.976 | -0.2222 | Yes |
| 206 | 100007048 | bcl2b |  |  | 29242 | -0.987 | -0.2173 | Yes |
| 207 | 445289 | kras |  |  | 29269 | -0.994 | -0.2121 | Yes |
| 208 | 403146 | akt2l |  |  | 29342 | -1.012 | -0.2083 | Yes |
| 209 | 573122 | tuba1c |  |  | 29368 | -1.020 | -0.2030 | Yes |
| 210 | 798445 | LOC798445 |  |  | 29435 | -1.044 | -0.1988 | Yes |
| 211 | 550475 | ctsk |  |  | 29445 | -1.045 | -0.1928 | Yes |
| 212 | 561006 | daxx |  |  | 29456 | -1.048 | -0.1868 | Yes |
| 213 | 561021\_1 | si:dkey-103e21.5 |  |  | 29577 | -1.086 | -0.1840 | Yes |
| 214 | 100001065 | si:ch211-165b19.5 |  |  | 29880 | -1.195 | -0.1864 | Yes |
| 215 | 567021\_1 | ern2 |  |  | 29910 | -1.206 | -0.1801 | Yes |
| 216 | 58133\_1 | ngfb |  |  | 29959 | -1.227 | -0.1742 | Yes |
| 217 | 373125 | aifm1 |  |  | 30075 | -1.274 | -0.1702 | Yes |
| 218 | 337730 | capn8 |  |  | 30080 | -1.277 | -0.1626 | Yes |
| 219 | 30590 | tp53 |  |  | 30325 | -1.401 | -0.1619 | Yes |
| 220 | 559384\_1 | prf1.5 |  |  | 30538 | -1.531 | -0.1594 | Yes |
| 221 | 559564 | eif2ak3 |  |  | 30600 | -1.590 | -0.1518 | Yes |
| 222 | 140621\_1 | casp3a |  |  | 30781 | -1.743 | -0.1470 | Yes |
| 223 | 246726 | birc5b |  |  | 30905 | -1.873 | -0.1396 | Yes |
| 224 | 57934 | actb1 |  |  | 31071 | -2.048 | -0.1325 | Yes |
| 225 | 564295\_2 | traf2a |  |  | 31089 | -2.074 | -0.1206 | Yes |
| 226 | 58100\_1 | badb |  |  | 31292 | -2.420 | -0.1124 | Yes |
| 227 | 431763\_1 | gadd45ab |  |  | 31309 | -2.449 | -0.0982 | Yes |
| 228 | 100000252\_1 | pik3r3b |  |  | 31434 | -2.733 | -0.0857 | Yes |
| 229 | 140621 | casp3a |  |  | 31691 | -3.652 | -0.0718 | Yes |
| 230 | 791531\_1 | casp6l1 |  |  | 31738 | -4.015 | -0.0491 | Yes |
| 231 | 552927\_1 | casp6 |  |  | 31754 | -4.166 | -0.0246 | Yes |
| 232 | 373107\_1 | birc2 |  |  | 31825 | -4.799 | 0.0021 | Yes |
Table: GSEA details [plain text format]

  

Fig 2: APOPTOSIS(DRE04210)      
 Blue-Pink O' Gram in the Space of the Analyzed GeneSet

  

Fig 3: APOPTOSIS(DRE04210): Random ES distribution      
 Gene set null distribution of ES for **APOPTOSIS(DRE04210)**
